# End-of-life preparedness and emotional suffering in patients and caregivers: Findings from an international cohort study spanning the period before and after death

**DOI:** 10.1177/02692163251405361

**Published:** 2026-01-13

**Authors:** Clément Meier, Verónica Inés Veloso, Bélen Carballo, Eva Víbora Martín, Pilar Barnestein-Fonseca, Dröfn Birgisdóttir, Valgerður Sigurðardóttir, Ida Korfage, Agnes van der Heide, Vilma A. Tripodoro

**Affiliations:** 1 The Faculty of Business and Economics (HEC), University of Lausanne, Switzerland; 2 Swiss Centre of Expertise in the Social Sciences (FORS), Instituto Pallium Latinoamérica, Ciudad de Buenos Aires, Argentina; 3 Instituto Pallium Latinoamérica, Buenos Aires, Ciudad de Buenos Aires, Argentina; 4 Instituto de Investigaciones Médicas Alfredo Lanari, Universidad de Buenos Aires, Ciudad de Buenos Aires, Argentina; 5 CUDECA Institute for Training and Research in Palliative Care, CUDECA Hospice Foundation, Málaga, Spain; 6 IBIMA Institute-Plataforma Bionand Group CA15: Palliative Care, Málaga, Spain; 7 The Institute for Palliative Care, Department of Clinical Sciences Lund, Lund University, Sweden; 8 Palliativt Utvecklingscentrum, Lund University & Region Skåne, Sweden; 9 Landspítali – The National University Hospital of Iceland, Reykjavík, Iceland; 10 Erasmus Medical Centre, Rotterdam, The Netherlands; 11 ATLANTES Global Observatory of Palliative Care, University of Navarra, Pamplona, Spain

**Keywords:** end-of-life preparedness, emotional suffering, patients, caregivers, end-of-life care, cohort study

## Abstract

**Background:**

Preparing for the end of life is believed to help mitigate emotional suffering for both patients and their caregivers. However, empirical evidence on the emotional benefits of feeling prepared for death remains limited.

**Aim:**

This study uses data from the international iLIVE project to examine how perceived end-of-life preparedness is associated with emotional suffering among patients and their caregivers before and after death.

**Design:**

Data from a prospective cohort study from the iLIVE project (2020–2023) were analyzed. Participants were surveyed at baseline, 1-month follow-up, and where possible after the patient’s death. The association between end-of-life preparedness (ICECAP-SCM/CPM) and emotional suffering (ICECAP-SCM/CPM & HGRC) was examined using OLS regression models, adjusting for socio-demographic and health-related covariates.

**Setting/participant:**

Data came from 1041 patients and 496 caregivers across 11 countries, enrolled in the iLIVE project.

**Results:**

Feeling fully prepared for the end of life was significantly associated with lower levels of emotional suffering for both patients and caregivers. Among patients, preparedness was linked to reduced emotional suffering at baseline and follow-up. For caregivers, these associations were even more pronounced at baseline, follow-up, and after the patient’s death.

**Conclusions:**

Perceived preparedness for the end of life was associated with lower emotional suffering for patients approaching death and their caregivers, both during the illness and after bereavement. These findings suggest that encouraging end-of-life planning may support emotional well-being across the final phase of life and beyond.

**What is already known about the topic?**

- End-of-life preparedness is thought to ease emotional suffering in patients and caregivers facing life-limiting conditions. Prior studies suggest that those who feel more prepared may experience less emotional suffering, but robust evidence remains scarce, particularly across international settings and among both patients and caregivers.

**What this paper adds?**

- This study provides multinational evidence that perceived end-of-life preparedness is significantly associated with lower emotional suffering among both patients and caregivers. Importantly, the protective association holds across different stages during the care trajectory and after bereavement, highlighting the potential association between perceived preparedness and lower emotional suffering over time.

**Implications for practice, theory, or policy**

- The findings emphasize the value of integrating end-of-life preparedness into routine palliative care. By addressing both the practical and emotional dimensions of readiness, healthcare professionals may help foster resilience and reduce suffering for patients and their families. Supporting subjective preparedness could be a considered as a potential component of anticipatory guidance and psychosocial interventions throughout the end-of-life journey.

## Introduction

The final phase of life is often accompanied by significant emotional challenges for both patients and those who care for them.^1,2^ Feelings of anxiety, distress, and emotional suffering are common as individuals encounter the reality of dying and the anticipated loss of a loved one .^3,4^ As such, strategies that may alleviate emotional suffering in this context are of increasing interest to clinicians, caregivers, and policymakers.^5^

One such strategy is enhancing end-of-life preparedness, a multifaceted concept that encompasses practical, emotional, and existential elements such as financial and legal arrangements, saying goodbye to loved ones, clarifying treatment preferences, and mentally preparing for death.^6–8^ It is widely presumed that when patients and families feel prepared for death, their emotional burden is eased, potentially facilitating a more peaceful dying process for the patient and a smoother transition into bereavement for the caregiver.^9,10^ Current evidence supports this assumption: for instance, better emotional preparedness in patients with life-limiting conditions has been associated with lower anxiety and depression near the end of life,^11,12^ and caregivers who feel more prepared for the death tend to experience less complicated grief after loss.^13–16^ Moreover, end-of-life preparedness, when encompassing both cognitive and emotional readiness, is associated with lower emotional suffering, better quality of life, greater use of hospice care, and reduced likelihood of receiving potentially inappropriate aggressive treatments at the end of life.^17^

However, while preparedness has been linked to emotional suffering, less is known about how it impacts on emotional suffering over time, particularly when measured before and after death. Few studies have examined both patients and caregivers concurrently, and even fewer have explored how perceived preparedness evolves over time and how these dynamics relate to emotional well-being throughout the caregiving and bereavement process.^18^ Empirical evidence for the emotional benefits of end-of-life preparedness remains limited and somewhat fragmented, particularly from prospective studies spanning multiple countries.

To address these gaps, this study uses data from the international iLIVE project, which followed patients with life-limiting conditions and their caregivers across 11 countries.^19^ The aim is to examine the associations between perceived end-of-life preparedness and emotional suffering at three key stages: (1) during the last months of life (baseline), (2) approximately 1 month later (follow-up), and (3) after the patient’s death from the caregiver’s perspective. By assessing both patients and caregivers across these stages, this study contributes to a more nuanced understanding of how perceived end-of-life preparedness may buffer emotional suffering and support psychological resilience throughout the end-of-life trajectory.

## Methods

### Research question

This research draws on data from the international iLIVE project (Live Well, Die Well; Horizon 2020, ID: 825731),^19,20^ which explores the needs and experiences of individuals with life-limiting conditions and their relatives. The central research question of this study is how perceived end-of-life preparedness is associated with emotional suffering among patients and caregivers before and after death.

### Design

Participants were enrolled into a prospective observational study conducted in various clinical care settings.^19^ Data were collected at three timepoints: baseline and follow-up (approximately 1 month later) for patients and caregivers, and an additional post-bereavement follow-up for caregivers where feasible.

### Setting

The study was conducted in 11 countries: Argentina, Switzerland, Germany, Spain, United Kingdom, Iceland, Netherlands, Norway, New Zealand, Sweden, and Slovenia.

### Population

Patients were eligible if they were aged 18 or older, aware of their life-limiting condition, and capable of providing informed consent.^19^ Individuals with significant cognitive or communication impairments were not included. In total, 1041 patients and 496 caregivers completed the baseline assessment; 537 patients and 299 caregivers were followed up, and 119 caregivers responded after the patient’s death.

### Sampling

Inclusion was based on clinical judgment, using an adapted version of the Gold Standards Framework Proactive Identification Guidance, a tool designed to help identify patients who may be in their final months of life, and when needed, supported by the Supportive and Palliative Care Indicators Tool, which provides clinical indicators of deteriorating health due to life-limiting conditions.^21^

### Recruitment

Patients were approached in clinical care settings between May 2020 and December 2023. Ethical approvals were secured in all participating locations.

### Data collection

The questionnaires assessed emotional suffering separately for patients and caregivers using standardized self-report items. For patients, it was measured using an item from the ICECAP-Supportive Care Measure (ICECAP-SCM),^22^ which asked: “*Please indicate which statement best describes your situation at this moment, by ticking one box per item: Emotional suffering – Experiencing worry or distress?*” Responses ranged from *rarely, sometimes, often*, to *always*. For analysis, responses were coded on a 4-point scale from 1 (*rarely*) to 4 (*always*), with higher values indicating greater emotional suffering. This measure was collected at both baseline and follow-up. For caregivers, emotional suffering was measured with the following question, adapted from the ICECAP-CPM instrument^23^: “*Thinking about your experience, please place a tick one box in each group below, to indicate which statement best describes your situation at the moment. Emotional distress to you, related to the condition of your relative. This includes things like being free from emotional distress resulting from: seeing your relative in pain or discomfort; seeing the loss of dignity, or a lack of respect given to your relative; seeing a lack of care and attention given to your relative.”* The five response options were: *free, mostly free, somewhat free, mostly not free*, and *not free* from emotional distress. These were coded from 1 to 5, with higher scores indicating greater emotional suffering. This measure was collected at baseline and follow-up. In addition, for caregivers who completed the questionnaire after the patient’s death, emotional suffering was assessed using an adapted version of the Grief Distress Score (Appendix 1), derived from the Hogan Grief Reaction Checklist (HGRC).^24^ This composite measure was calculated by averaging responses to 13 items covering domains such as despair, emotional pain, blame, and psychological disorganization. Each item was rated on a 5-point Likert scale ranging from 1 (*does not describe me at all*) to 5 (*describes me very well*), with higher scores indicating greater emotional suffering. Items reflecting post-bereavement personal growth were excluded from the composite score in order to focus specifically on distress-related aspects of grief.

The questionnaires assessed end-of-life preparedness separately for patients and caregivers using standardized self-report items from the ICECAP-SCM.^22,23^ For patients, it asked: “*Please indicate which statement best describes your situation at this moment, by ticking one box per item: Being prepared – Having financial affairs in order, having your funeral planned, saying goodbye to family and friends, resolving things that are important to you, having treatment preferences in writing or making a living will”* (Appendix 2). Response options were *not any, few, some*, and *most* of the preparations I want to make. For analysis, participants who selected *most* were categorized as fully prepared (coded 1), and those who selected *not any, few*, or *some* were categorized as not fully prepared (coded 0). This measure was included at both baseline and follow-up. Caregivers were asked a similar question: “*Thinking about your experience, please place a tick one box in each group below, to indicate which statement best describes your situation at the moment. Preparing and coping. This includes things like being prepared for deterioration of your relative’s health; having your relative’s post-bereavement affairs in order; being free from guilt and regrets”* (Appendix 3). The response categories were *fully able, mostly able, somewhat able, mostly unable*, and *completely unable* to prepare for and cope with deterioration of my relative’s health. Caregivers who selected *fully able* were considered fully prepared (coded 1), while all others were coded as not fully prepared (coded 0). This measure was assessed at baseline and follow-up.

All statistical models were adjusted for individual characteristics, the variables included in the models differed slightly for patients and caregivers. For patients the models included: age (continuous), gender (male, female, or other), living situation at baseline (with relatives, alone, or in an institution), education level (none/primary, secondary, or tertiary/university), main diagnosis of the patient (cancer, cardiovascular disease, neurological disease, pulmonary disease, or frailty due to old age and other conditions), self-rated health at baseline or follow-up using a 5-point Likert scale recoded in three categories (poor, fair, or good/very good/excellent) and country. For caregivers the models included: age (continuous), gender (male, female, or other), living situation at baseline (with relatives, alone, or in an institution), relation to patient (my partner, my parent, other), education level (none/primary, secondary, or tertiary/university), main diagnosis of the patient cared for (same categories as above), caregiver health score at baseline, follow-up, or after death using a Visual Analog Scale ranging from 0 (worst imaginable health) to 100 (best imaginable health), and country.

### Statistical analysis

Demographic, socio-economic and health characteristics of the study participants are describe using numerical counts. Bivariate associations between end-of-life preparedness and emotional suffering of the patients and caregivers are presented using bar charts. Multivariable ordinary least squares (OLS) regression models were used to assess the associations between end-of-life preparedness and emotional suffering at baseline, follow-up, and, among caregivers, after the patient’s death. All models adjusted for the aforementioned covariates. To ensure transparency, full model estimates including all covariates are reported in Tables 2 and 3, although these are not the primary focus of interpretation. Results are reported as average marginal effects (AMEs) with robust standard errors. Statistical significance was set at *p* < 0.05 (two-tailed). Analyses were conducted using STATA/SE 18.0 (StataCorp, College Station, TX).

## Results

Table 1 summarizes the demographic, health, and care characteristics of patients and caregivers. Patients averaged 71.1 years and caregivers 58.9 years at baseline. While patient gender was evenly distributed, most caregivers were women (74.6%). At baseline, most patients lived with relatives (60.9%), and the majority of caregivers also lived with relatives (87.9%). Over half of caregivers were caring for a partner (51.6%) and more than a third for a parent (34.7%), while 13.7% supported another relative or non-family member. Over half of both patients and caregivers had tertiary education. The most common diagnosis among patients was cancer (78.2%), followed by pulmonary (8.5%), cardiovascular (7.8%), and neurological diseases (3.7%), with a small proportion affected by age-related frailty (2.7%). Patients mainly rated their health as fair (40.2%) or poor (35.5%), while caregivers’ self-rated health was higher but declined slightly at follow-up. Emotional suffering varied, with 37.1% of patients and 27.5% of caregivers reporting low levels. Post-loss, caregivers’ mean grief distress score was 2.2 (SD = 0.8). Preparedness for the end of life was reported by 64.9% of patients and 25.0% of caregivers at baseline. At follow-up, 64.1% of patients and 25.4% of caregivers reported feeling fully prepared.

**Table 1.** Characteristics of the study population, patients and caregivers in the last phase of life, iLIVE, 2020/2023.

	Patients		Caregivers		
	Baseline (1041)	Follow-up (537)	Baseline (496)	Follow-up (299)	After patient’s death (119)
Age, mean (SD)	71.1 (12.3)	71.1 (12.3)	58.9 (14.2)	59.2 (14.2)	58.4 (14.9)
Gender					
Men	536	270	126	80	25
Women	503	267	370	219	94
Other	2	0	0	0	0
Living situation at baseline					
With relatives	635	330	436	262	104
Alone	293	130	59	37	15
In an institution	113	77	1	0	0
Relation to patient					
My partner			256	155	61
My parent			172	99	40
Other			68	45	18
Education level					
None/primary	157	75	51	35	14
Secondary	325	165	146	88	41
Tertiary/University	559	297	299	176	64
Main diagnosis					
Cancer	814	388	400	231	103
Cardiovascular disease	81	57	33	19	6
Neurological disease	38	30	21	19	6
Pulmonary disease	80	46	32	19	2
Frailty due to old age and other	28	16	10	11	2
Self-rated heath patients					
Poor	370	149			
Fair	418	232			
Good/Very good/Excellent	253	156			
Self-rated heath score caregivers			73.6 (18.3)	71.9 (18.1)	81.8 (77.6)
Countries					
	Argentina (190), Switzerland (76), Germany (66), Spain (62), UK (66), Iceland (95), Netherlands (102), Norway (136), New Zealand (41), Sweden (51), Slovenia (156)	Argentina (103), Switzerland (28), Germany (34), Spain (40), UK (19), Iceland (26), Netherlands (50), Norway (79), New Zealand (32), Sweden (21), Slovenia (105)	Argentina (136), Switzerland (19), Germany (15), Spain (74), UK (11), Iceland (71), Netherlands (25), Norway (63), New Zealand (35), Sweden (18), Slovenia (29)	Argentina (78), Switzerland (11), Germany (10), Spain (62), UK (7), Iceland (15), Netherlands (18), Norway (44), New Zealand (29), Sweden (12), Slovenia (13)	Argentina (36), Switzerland (1), Germany (4), Spain (40), Iceland (5), Netherlands (5), Norway (9), New Zealand (11), Sweden (7), Slovenia (1)
Emotional suffering patients					
Rarely	386	191			
Sometimes	380	206			
Often	186	108			
Always	89	32			
Emotional suffering caregivers					
Free			33	17	
Mostly free			103	65	
Somewhat free			179	114	
Mostly not free			116	64	
Not free			65	39	
Grief distress score					2.2 (0.8)
Fully prepared for end of life					
No	366	193	372	223	88
Yes	675	344	124	76	31

*Note.* Number of observations for the whole sample at baseline, follow-up, and after patient’s death.

**Figure 1.**
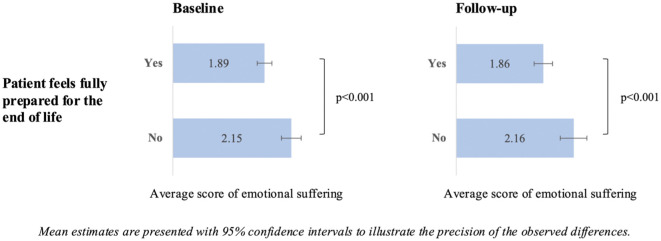
Emotional suffering and end-of-life preparedness among patients during the last phase of life, iLIVE, 2020/2023.

Figure 1 illustrates bivariate associations between emotional suffering and end-of-life preparedness among patients during the final phase of life. At both baseline and follow-up, patients who reported feeling fully prepared for the end of life experienced significantly lower emotional suffering compared to those who did not. At baseline, the average emotional suffering score was 1.89 among fully prepared patients, compared to 2.15 among those not fully prepared (*p* < 0.001). A similar pattern was observed at follow-up, with average scores of 1.86 and 2.16, respectively (*p* < 0.001).

**Figure 2.**
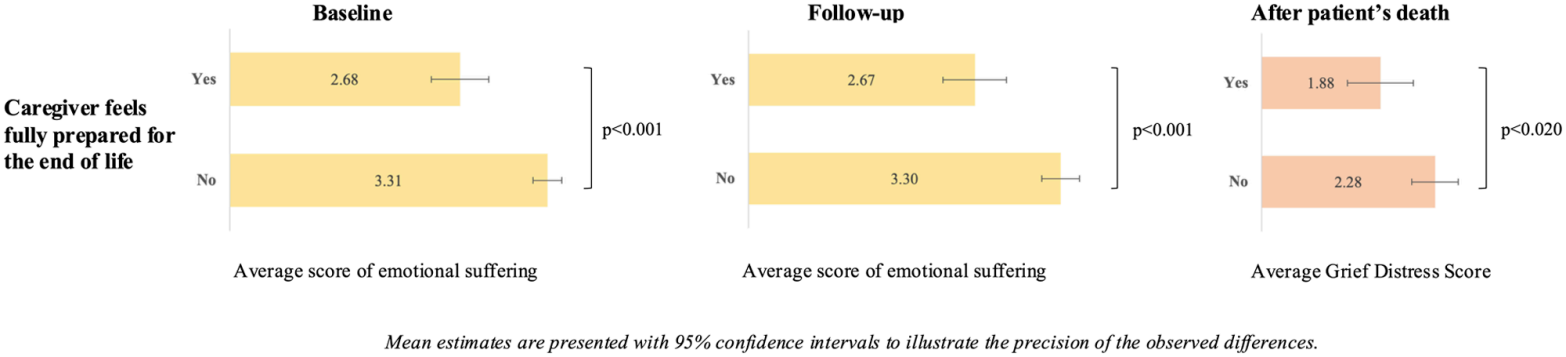
Emotional suffering and end-of-life preparedness among caregivers at baseline, follow-up, and after patients’ death, iLIVE, 2020/2023.

Bivariate associations between emotional suffering and end-of-life preparedness among caregivers during the final phase of life and after the patient’s death are presented in Figure 2. At baseline, follow-up, and after the patient’s death, caregivers who reported feeling fully prepared experienced significantly less emotional suffering than those who did not. At baseline, the average emotional suffering score was 2.68 among fully prepared caregivers, compared to 3.31 among those not fully prepared (*p* < 0.001). This pattern remained consistent at follow-up, with scores of 2.67 for the prepared group and 3.30 for those who did not feel fully prepared (*p* < 0.001). In addition, caregivers who reported feeling fully prepared for the end of life had significantly lower grief distress scores than those who did not. The average grief distress score was 1.88 among fully prepared caregivers, compared to 2.28 among those who were not fully prepared (*p* = 0.020).

The adjusted associations between end-of-life preparedness and emotional suffering among patients are presented in Table 2, based on OLS regression models adjusting for age, gender, living situation at baseline, education level, main diagnosis, self-rated health, and country. At both baseline and follow-up, patients who reported feeling fully prepared for the end of life experienced significantly lower levels of emotional suffering. Specifically, being fully prepared was associated with an average reduction in emotional suffering at baseline (AME = −0.17, *p* < 0.01) and at follow-up (AME = −0.21, (*p* < 0.01).

**Table 2.** Partial association between patients’ emotional suffering and end-of-life preparedness adjusted for covariates at baseline and follow-up, iLIVE, 2020–2023.

	Emotional suffering	Emotional suffering
	Baseline	Follow-up
Age	−0.01** (0.00)	−0.00 (0.00)
Gender (men)		
Women	0.06 (0.06)	0.21** (0.08)
Other	1.30*** (0.10)	-
Living situation at baseline (with relatives)		
Alone	0.02 (0.07)	0.04 (0.10)
In an institution	0.06 (0.11)	0.07 (0.16)
Education level (none/primary)		
Secondary	0.06 (0.10)	−0.07 (0.13)
Tertiary/University	0.03 (0.09)	−0.14 (0.12)
Main diagnosis (cancer)		
Cardiovascular disease	0.12 (0.12)	−0.07 (0.15)
Neurological disease	0.09 (0.19)	0.03 (0.19)
Pulmonary disease	0.26* (0.11)	0.16 (0.14)
Frailty due to old age and other	0.35 (0.20)	−0.20 (0.23)
Self-rated heath patients (poor)		
Fair	−0.29*** (0.07)	−0.59*** (0.10)
Good/Very good/Excellent	−0.57*** (0.07)	−0.77*** (0.10)
Countries (Argentina)		
Switzerland	−0.47*** (0.12)	−0.14 (0.18)
Germany	−0.53*** (0.15)	−0.29 (0.20)
Spain	0.04 (0.16)	−0.16 (0.19)
UK	−0.21 (0.14)	−0.45* (0.22)
Iceland	−0.38** (0.12)	−0.49* (0.19)
Netherlands	−0.19 (0.11)	−0.12 (0.14)
Norway	−0.29** (0.11)	−0.34* (0.13)
New Zealand	−0.26 (0.13)	−0.10 (0.17)
Sweden	−0.34** (0.13)	−0.23 (0.23)
Slovenia	−0.43*** (0.11)	−0.39** (0.14)
Fully prepared for end of life (no)		
Yes	−0.17** (0.06)	−0.21** (0.08)
Observations	1041	537

The table shows average marginal effects with standard errors in parentheses. Statistical significance: *p < 0.05, **p < 0.01, *** *p* < 0.001. The columns present the results from OLS regressions of average emotional suffering scores on end-of-life preparedness, adjusting for covariates at baseline and follow-up. The covariates include age, gender, living situation at baseline, education level, main diagnosis, self-rated health and countries.

Table 3 displays the adjusted associations between end-of-life preparedness and emotional suffering among caregivers, based on OLS regression models at baseline, follow-up, and after the patient’s death. All models were adjusted for caregiver age, gender, living situation at baseline, relation to patient, education level, self-rated health, the patient’s main diagnosis, and country. At baseline, caregivers who reported feeling fully prepared for the end of life had significantly lower emotional suffering scores (AME = –0.59, *p* < 0.001). This association remained consistent at follow-up (AME = –0.60, *p* < 0.001) and was also observed after the patient’s death (AME = −0.42, *p* < 0.05).

**Table 3.** Partial association between caregivers’ emotional suffering and end-of-life preparedness adjusted for covariates at baseline, follow-up and after patients’ death, iLIVE, 2020–2023.

	Emotional suffering	Emotional suffering	Emotional suffering
	Baseline	Follow-up	After patient’s death
Age	−0.00 (0.00)	−0.00 (0.01)	−0.00 (0.01)
Gender (men)			
Women	0.10 (0.10)	0.03 (0.13)	−0.09 (0.20)
Other	-	-	-
Living situation at baseline (with relatives)			
Alone	0.18 (0.15)	0.12 (0.18)	0.18 (0.21)
In an institution	0.47* (0.19)	-	-
Relation to patient (My partner)			
My parent	−0.09 (0.13)	0.18 (0.16)	−0.25 (0.23)
Other	−0.34* (0.14)	−0.22 (0.17)	0.05 (0.26)
Education level (none/primary)			
Secondary	0.28 (0.20)	0.31 (0.22)	0.24 (0.30)
Tertiary/University	0.32 (0.20)	0.08 (0.22)	0.07 (0.28)
Main diagnosis (cancer)			
Cardiovascular disease	−0.32 (0.19)	−0.09 (0.20)	−0.34 (0.29)
Neurological disease	−0.39 (0.26)	−0.24 (0.22)	0.12 (0.27)
Pulmonary disease	0.36 (0.22)	0.28 (0.29)	−0.60 (0.47)
Frailty due to old age and other	−0.26 (0.37)	0.41 (0.32)	0.53 (0.49)
Self-rated heath score caregivers	−0.01** (0.00)	−0.01* (0.00)	−0.00 (0.00)
Countries (Argentina)			
Switzerland	−0.58* (0.23)	0.05 (0.29)	−0.57* (0.22)
Germany	−0.77* (0.31)	−0.65 (0.33)	−0.12 (0.27)
Spain	0.07 (0.16)	0.07 (0.20)	0.33 (0.20)
UK	−0.19 (0.24)	−0.08 (0.33)	- -
Iceland	−0.76*** (0.16)	0.13 (0.31)	0.16 (0.30)
Netherlands	−0.62*** (0.17)	−0.42 (0.25)	0.06 (0.31)
Norway	−0.57*** (0.17)	−0.30 (0.22)	−0.14 (0.34)
New Zealand	−0.55*** (0.16)	−0.74*** (0.18)	0.60* (0.27)
Sweden	−1.37*** (0.22)	−0.91** (0.30)	−0.19 (0.25)
Slovenia	−0.55* (0.26)	−0.37 (0.33)	1.57*** (0.21)
Fully prepared for end of life (no)			
Yes	−0.59*** (0.11)	−0.60*** (0.14)	−0.42* (0.16)
Observations	496	299	119

The table shows average marginal effects with standard errors in parentheses. Statistical significance: *p < 0.05, **p < 0.01, *** *p* < 0.001. The columns present the results from OLS regressions of caregivers average emotional suffering scores on end-of-life preparedness, adjusting for covariates at baseline, follow-up, and after patient’s death. The covariates include age, gender, living situation at baseline, relation to patient, education level, main diagnosis of the patient, self-rated health and countries.

## Discussion

### Main findings/results of the study

This study provides multi-country evidence that perceived end-of-life preparedness is consistently associated with lower emotional suffering among both patients and caregivers. More precisely, we found that individuals who felt fully prepared for the end of life reported significantly lower levels of emotional suffering, not only during the care trajectory but also after death, during bereavement. These associations held even after adjusting for key sociodemographic and health-related factors, underscoring the robustness and potential clinical relevance of perceived preparedness. While the absolute differences were numerically small, they likely represent meaningful changes in perceived emotional suffering, as each step on the scale denotes a substantial qualitative shift in the person’s emotional state. In the context of terminal illness, even modest reductions in distress can translate into important gains in perceived well-being and quality of dying.

### What this study adds

For patients, feeling fully prepared was associated with lower emotional suffering at both baseline and follow-up, suggesting that readiness for death may support emotional well-being in the final months of life. These findings are in line with prior research showing that patients who engage in advance care planning or express a readiness to die report greater peace and less anxiety as death approaches.^25,26^ However, this study adds new insights by quantifying the association in a large, diverse, and prospective cohort, using a harmonized measure of emotional suffering across settings and timepoints. Among caregivers, the associations were even more pronounced. Those who felt fully prepared to cope with the patient’s deterioration experienced substantially lower emotional suffering, both before and after the patient’s death. This is consistent with previous studies indicating that caregiver preparedness for death is linked to better mental health outcomes, including lower levels of depression, distress, and grief intensity.^27–29^ Importantly, recent longitudinal evidence shows that preparedness is dynamic and does not necessarily increase over time without targeted support.^30^ This suggests that fostering preparedness may help reduce emotional suffering over time and could serve as a key target for supportive interventions aimed at improving emotional wellbeing among caregivers. Although bereavement support is often delivered reactively, our findings underscore the value of anticipatory guidance and emotional preparation during the caregiving phase. A systematic review on anticipatory grief highlights how addressing emotional needs before death can alleviate distress in bereavement.^31^ Taken together, these results support the hypothesis that end-of-life preparedness may act as a protective factor against emotional suffering across the continuum of dying, caregiving, and grieving.

However, when interpreting these findings, it should be taken into account that perceived preparedness may in part reflect the effects of multidimensional palliative care interventions rather than solely an individual’s capacity. Given that some participants in our study were followed in specialized care settings, it is plausible that their sense of preparedness was shaped or enhanced by the support they received. This has practical implications for clinical care, suggesting that healthcare professionals should not only facilitate logistical end-of-life planning but also attend to patients’ and families’ felt sense of readiness. In addition, since emotional preparedness is distinct from prognostic awareness, it is essential to go beyond simply providing information and actively support emotional readiness through relational care, compassionate dialog, and tailored psychosocial interventions.^32^

### Strengths and weaknesses/limitations of the study

This study has several limitations. First, all variables were self-reported, which may lead to reporting bias. While participants with significant cognitive or communication impairments were excluded from the study, some inaccuracies may still occur due to emotional strain, fatigue, or distress experienced during the end-of-life context, potentially affecting recall or interpretation of questions. Then, although the items were derived from validated instruments (ICECAP-SCM, ICECAP-CPM, HGRC), emotional suffering was assessed using single items at each timepoint or composite scores with limited psychometric evaluation in our sample, which may limit measurement depth, precision, and reproducibility in future studies. Nonetheless, consistency across patient and caregiver samples supports the observed associations. Furthermore, preparedness was assessed using a single-item measure reflecting a global perception, limiting our ability to disentangle its emotional, practical, and informational components and their distinct associations with emotional suffering. In addition, although worded differently for patients (being prepared) and caregivers (preparing and coping), both items are based on the ICECAP framework and reflect similar dimensions of subjective end-of-life readiness.^23,33^ Moreover, while the longitudinal design is a strength, both selective attrition and variability in follow-up may have introduced bias. Some participants may have discontinued participation due to declining health, emotional distress, or death, which could result in underrepresentation of individuals experiencing greater suffering. Additionally, differences in the timing and completeness of follow-up across sites, despite standardized protocols, may have led to inconsistencies affecting comparability of responses over time. In addition, because some participants were recruited in palliative care settings, access to specialized support may have enhanced both preparedness and well-being. Lastly, the observational design and constraints of secondary data, including attrition and missingness, limited our ability to pursue robust causal modeling. Thus, it is possible that individuals with better emotional well-being feel more prepared, rather than preparedness leading to lower suffering. However, this reverse association is not strongly supported by the existing literature, which generally suggests that preparedness precedes and contributes to improved psychological outcomes for both patients and caregivers. Moreover, cultural differences in attitudes toward death and preparedness were not formally explored due to limited sample sizes in several countries, but they may influence both the perception of preparedness and its emotional correlates. Future research with larger and more balanced country-level samples should examine how cultural context shapes end-of-life experiences and modifies these associations.

## Conclusion

In this exploratory study, we found that perceived preparedness for the end of life was consistently associated with lower emotional suffering among patients in the final months of life and their caregivers, both during the caregiving period and after bereavement. Individuals who report feeling fully prepared consistently experienced significantly less emotional suffering across key stages of the end-of-life trajectory. These findings highlight the potential of end-of-life preparedness as a meaningful and modifiable protective factor for emotional well-being. Importantly, our results underscore that preparedness is not solely a practical or logistical concern but also reflects emotional and psychological readiness that may influence how individuals cope with dying and loss. As such, integrating strategies that enhance perceived preparedness into routine palliative care, through anticipatory guidance, clear communication, and support for planning may offer critical benefits not only for patients’ peace of mind, but also for caregivers’ resilience in the face of loss.

## Appendices

**Appendix 1.** Hogan Grief Reaction Checklist.

Now you will see of a list of thoughts and feelings that you may have had since your relative died. Please read each statement carefully and choose the number that best describes the way you have been feeling during the past 2 weeks, including today. Circle the number that best describes you. *1 = Does not describe me at all, 2 = Does not quite describe me, 3 = Describes me fairly well, 4 = Describes me well, 5 = Describes me very well* a) My hopes are shattered b) I have learned to cope better with life c) I have little control over my sadness d) I feel like I am in shock e) I feel as though I am a better person f) I believe I should have died and he or she should have lived g) I have a better outlook on life h) I feel heaviness in my heart i) I want to die to be with him or her j) I have more compassion for others k) I am stronger because of the grief I have experienced l) I don’t believe I will ever be happy again m) I agonize over his or her death n) I am a more forgiving person o) I feel like I am walking in my sleep p) I am more tolerant of myself q) I have difficulty accepting the permanence of the death r) I am more tolerant of others s) I have hope for the future t) I feel hopeless u) I reached a turning point where I began to let go of some of my grief v) I frequently cry w) I ache with loneliness x) I am having more good days than bad y) I care more deeply for others

**Appendix 2.** Patients’ end-of-life preparedness – ICECAP-SCM.

Please indicate which statement best describes your situation AT THIS MOMENT, by ticking one box per item: Being prepared – Having financial affairs in order, having your funeral planned, saying goodbye to family and friends, resolving things that are important to you, having treatment preferences in writing or making a living will *1 = I have not had the opportunity to make any of the preparations I want to make* *2 = I have had the opportunity to make a few of the preparations I want to make* *3 = I have had the opportunity to make some of the preparations I want to make* *4 = I have had the opportunity to make most of the preparations I want to make*

**Appendix 3.** Caregivers’ end-of-life preparedness – ICECAP-SCM.

Thinking about your experience, please place a tick ONE box in EACH group below, to indicate which statement best describes your situation at the moment. Preparing and coping. This includes things like: - being prepared for deterioration of your relative’s health; - having your relative’s post-bereavement affairs in order; - being free from guilt and regrets. *1 = I have been completely unable to prepare for and cope with deterioration of my relative’s health* *2 = I have been mostly unable to prepare for and cope with deterioration of my relative’s health* *3 = I have been somewhat able to prepare for and cope deterioration of my relative’s health* *4 = I have been mostly able to prepare for and cope with deterioration of my relative’s health* *5 = I have been fully able to prepare for and cope with deterioration of my relative’s health*
